# A Deep Insight into the Sialome of Male and Female *Aedes aegypti* Mosquitoes

**DOI:** 10.1371/journal.pone.0151400

**Published:** 2016-03-21

**Authors:** José M. C. Ribeiro, Ines Martin-Martin, Bruno Arcà, Eric Calvo

**Affiliations:** 1 Laboratory of Malaria and Vector Research, Section of Vector Biology, National Institute of Allergy and Infectious Diseases, Bethesda, Maryland, United States of America; 2 Department of Public Health and Infectious Diseases, Parasitology Section, Sapienza University of Rome, Roma, Italy; New Mexico State University, UNITED STATES

## Abstract

Only adult female mosquitoes feed on blood, while both genders take sugar meals. Accordingly, several compounds associated with blood feeding (i.e. vasodilators, anti-clotting, anti-platelets) are found only in female glands, while enzymes associated with sugar feeding or antimicrobials (such as lysozyme) are found in the glands of both sexes. We performed *de novo* assembly of reads from adult *Aedes aegypti* female and male salivary gland libraries (285 and 90 million reads, respectively). By mapping back the reads to the assembled contigs, plus mapping the reads from a publicly available *Ae*. *aegypti* library from adult whole bodies, we identified 360 transcripts (including splice variants and alleles) overexpressed tenfold or more in the glands when compared to whole bodies. Moreover, among these, 207 were overexpressed fivefold or more in female vs. male salivary glands, 85 were near equally expressed and 68 were overexpressed in male glands. We call in particular the attention to C-type lectins, angiopoietins, female-specific Antigen 5, the 9.7 kDa, 12–14 kDa, 23.5 kDa, 62/34 kDa, 4.2 kDa, proline-rich peptide, SG8, 8.7 kDa family and SGS fragments: these polypeptides are all of unknown function, but due to their overexpression in female salivary glands and putative secretory nature they are expected to affect host physiology. We have also found many transposons (some of which novel) and several endogenous viral transcripts (probably acquired by horizontal transfer) which are overexpressed in the salivary glands and may play some role in tissue-specific gene regulation or represent a mechanism of virus interference. This work contributes to a near definitive catalog of male and female salivary gland transcripts from *Ae*. *aegypti*, which will help to direct further studies aiming at the functional characterization of the many transcripts with unknown function and the understanding of their role in vector-host interaction and pathogen transmission.

## Introduction

Saliva of blood sucking animals contains a diverse cocktail of pharmacologically active components that counteract their hosts’ physiological responses against blood loss (hemostasis) as well as have immunomodulatory and anti-inflammatory properties [1]. Perhaps due to these activities, saliva also affects transmission of pathogens, including arboviruses [2–11]. In the case of mosquitoes, only the adult female feeds on blood which is necessary for egg development in non-autogenous organisms. Both adult genres will also feed sugary solutions, which provide energy for flight and basic metabolic needs [12]. Sugar feeding is also assisted by salivation, which provides a liquid medium to dilute solid sugars [13], enzymes for digesting complex sugars [14, 15] and antimicrobial peptides, such as lysozyme [16], that presumably help to control microbial growth in the mosquito crop where the sugar meals are stored. The anatomy of the adult salivary glands (SG) in mosquitoes reflects this sexual dimorphism. The male salivary gland (MSG) is miniscule compared to the female organ, which has additional large distal-lateral lobes and a medial lobe. The proximal-lateral lobes of the female salivary glands (FSG) are homologous to MSG, while components assisting blood feeding are synthesized and stored in the female-only lobes. Indeed glycosidases assisting sugar feeding are found in the female proximal-lateral lobes, while the anti-platelet apyrase enzyme is located in the distal-lateral lobes [17–19], and the vasodilator sialokinin is transcribed in the medial lobe [20–22]. Previous sialotranscriptomics of *Aedes aegypti* identified near 100 putative secreted proteins [23, 24] and in one of these studies [24] the tissue specificity transcription for 71 gene products were evaluated by RT-PCR. Moreover, an RNA “in situ” hybridization (ISH) study reported fine spatial distributions in the *Ae*. *aegypti* tri-lobed gland of 30 salivary gland transcripts, with 12 of them exhibiting proximal-lateral lobe-specific accumulations and the remaining 18 showing distal-lateral and/or medial lobe-specific transcription [25]. Finally, a microarray approach using Affymetrix chips compared gender-specific gene expression in several organs of *Anopheles gambiae* mosquitoes, including male and female salivary glands [26].

In the present work we performed “de novo” assembly of Illumina-derived reads from cDNA libraries of both adult male and female *Ae*. *aegypti* salivary glands. We mapped the library reads back to the assembly, allowing determination of male- and female-specific transcripts. We also used a publicly available whole body (WB) transcriptome from both male and female mosquitoes [27], allowing for identification of salivary (unrelated to sex) enriched transcripts. Novel transcripts were identified, and many known partial coding sequences were extended. This work represents the first transcriptome study on adult *Ae*. *aegypti* MSG and it contributes to a near definitive catalog of male and female salivary gland transcripts from *Ae*. *aegypti*, which will help to direct further studies aiming at the functional characterization of the many transcripts with unknown function, as well as at a deeper understanding of their role in vector-host interaction and arboviral transmission.

## Materials and Methods

### Mosquitoes

*Aedes aegypti* (Liverpool strain) mosquitoes were reared in standard insectary conditions at Laboratory of Malaria and Vector Research, National Institute of Allergy and Infectious Diseases (28 C, 80% humidity, with a 12-h light/dark cycle and maintained with 10% Karo syrup solution) under the expert supervision of Mr. Andre Laughinghouse. Sugar-fed adult mosquitoes (2- to 5-days old) were anesthetized with CO_2_, transferred to an ice-chilled plate, and their salivary glands dissected under a stereomicroscope in sterile 25 mM Tris-HCl and 150 mM NaCl at pH 7.4 and immediately transferred to an Eppendorf tube containing 200 μl of RNAlater (ThermoFisher Scientific) solution. Pools of salivary glands (50 pairs from females and 150 from males) were kept overnight at 4°C and then stored at −80°C until used for RNA extraction. For quantitative PCR experiments, pools of 15 or 30 pairs of SG were respectively collected from female or male adults and transferred to 100 μl of Trizol reagent (Life Technologies). Samples were kept at -80°C until processing.

### RNA preparation

The mRNA from mosquito salivary glands was isolated using the FastTrack MAG Micro mRNA Isolation kit (LifeTechnologies, Carlsbad, CA) according to the manufacturer instructions. Isolated mRNA integrity and concentration were analyzed with the Agilent Bioanalyzer 2100 using an Agilent RNA 6000 Nano Chip (Agilent Technologies, USA).

### cDNA library constructions and next generation sequencing

The salivary gland mRNA library construction and sequencing were done by the NIH Intramural Sequencing Center. The SG library was constructed using the TruSeq RNA sample prep kit, v2 (Illumina Inc., San Diego, CA) following the manufacturer recommendations. The resulting cDNA was fragmented using a Covaris E210 system (Covaris, Woburn, MA). Library amplification was performed using eight cycles to minimize the risk of over-amplification. Sequencing was performed on a HiSeq 2000 (Illumina) with v3 flow cells and sequencing reagents. The two prepared Illumina cDNA libraries were pooled to have three times as more female than male molar amounts, to have more female-derived reads in the expected more complex library. The resulted pooled cDNA library was run on a single lane of Illumina using a paired-end protocol. The read length obtained was of 101 nucleotides (nt).

### qPCR

Concentrations and OD_260/280_ ratios of nucleic acids were determined with the Nanodrop ND-1000 spectrophotometer. 1 μg of RNA was converted to cDNA by using the QuantiTect Reverse Transcripase Kit (Qiagen). Specific primers were designed to amplify suitable amplicons for qPCR (S1 Table). A qPCR mixture was prepared with SsoAdvanced Universal SYBR Green Supermix (Bio-Rad), 300 nM of each primer and 100 ng of cDNA. The cycling conditions were 95°C for 5 min, 40 cycles at 95°C for 10 s, 55°C for 30 s and 72°C for 20 s. Relative abundance of genes of interest was analyzed in a CFX96 Real-Time thermal cycler and normalized against *A*. *aegypti* 40S ribosomal protein S7 transcript (AAEL009496-RA) as the reference gene. Three biological replicates were used and all samples were tested in duplicates. Non-template controls were included in all qPCR experiments as negative controls. Single melt curves of each amplicon were checked for specificity validation. qPCR data were manually inspected and analyzed with the Bio-Rad CFX Manager 3.1. The fold change for each target were calculated as 2^−ΔΔCt^ using the estimated ΔΔCt value ± standard error.

### Bioinformatic analysis

Bioinformatic analyses were conducted following methods described previously [28, 29]. Briefly, the fastq files were trimmed of low quality reads (<13) and concatenated for single-ended assembly using the Abyss [30] and SoapdenovoTrans [31] assemblers using k parameters from 21–91 in 5 fold increments. The combined fasta files plus the *Ae*. *aegypti* coding sequences (version 3.3) deposited in VectorBase [32] were further assembled using a iterative blast and CAP3 pipeline as previously described [33]. Coding sequences (CDS) were extracted based on the existence of a signal peptide in the longer open reading frame (ORF) and by similarities to other proteins found in the Refseq invertebrate database from the National Center for Biotechnology Information (NCBI), proteins from Diptera deposited at NCBI’s Genbank and from SwissProt. To obtain relative expression data to tissues other than salivary glands into the assembled transcriptome, we downloaded from the Sequence Read Archives (SRA) of the NCBI the reads from bioproject PRJNA261799 referring to whole body RNASeq data from sugar-fed, virgin male and female *Ae*. *aegypti* (Liverpool strain) [27]. This project was registered on 23/09/2014 and the reads made public on 05/21/2015. Reads for each library were mapped on the deducted CDS using blastn with a word size of 25, 1 gap allowed and 95% identity or better required. Up to five matches were allowed if and only if the scores were the same as the largest score. A Χ^2^ test was performed for each CDS to detect statistically significant differences between the number of reads in paired comparisons. Bonferroni and the false discovery rate (FDR) corrections of Benjamini & Hockberg [34] were done using the p.adjust program from the stats package version 3.3.0 which is part of the core R package [35]. The results of these tests are mapped to hyperlinked excel sheets presented as S1 File following column HN on worksheet named “Assembly”. The normalized ratio of the reads for paired comparisons was calculated as r1 x R2 / [R1 x (r2 +1)] and r2 x R1 / [R2 x (r1 +1)] where r1 and r2 are reads for libraries 1 and 2, and R1 and R2 are total number of reads from libraries 1 and 2 mapped to all CDS. One was added to the number of reads in the denominator to avoid division by zero. To compare transcript relative expression among contigs, we use the “expression index” (EI) defined as the number of reads mapped to a particular CDS multiplied by 100 and divided by the largest found number of reads mapped to a single CDS [28], which in the case of the FSG transcriptome was a value of 3,890,757 mapped to a long D7 protein CDS, and thus having an EI = 100. Reads per thousand nucleotides per million reads (RPKM) [36] and transcripts per million (TPM) for the four mapped libraries were calculated and mapped to the spreadsheet [37]. We calculated library pairwise TPM ratios, such as for female salivary gland and male whole body (MWB) libraries (FSG/MWB). The denominators of these ratios were always added of 0.1 to avoid division by zero. We used TPM for the comparisons of gene expression between libraries, but for the absolute values of expression we used the more conventional RPKM values, or normalized read ratios as defined above. Heatmap graphs were done with the program heatmap2 from the gplots package running within R package with default parameters and using Z scores for data normalization [38].

### Data availability

The raw Illumina fastq data were submitted to the Sequence Read Archive of the NCBI under bioproject PRJNA298896, biosample SAMN04168647 and runs SRR2659965 (female SG) and SRR2659966 (male SG). Extracted CDS were submitted to the Transcriptome Shotgun Annotation (TSA) portal of the NCBI under the accession GDUN00000000. The version described in this paper is the first version, GDUN01000000.

## Results and Discussion

### General aspects of the assembly

After removal of Illumina primers and trimming of low quality bases, we obtained 285,950,466 reads for the FSG and 89,786,274 reads for the MSG libraries, with an average length and median of 101 nt. These reads were assembled together using Abyss and SoapdenovoTrans with various kmer size parameters. The resulting assemblies plus the v.3.3 CDS of *Ae*. *aegypti* were then assembled together using a pipeline of iterative and parallelized blastn and cap3 where blastn with decreasing word sizes (from 300 to 60) fed the cap3 assembler through 15 iterations (S1 File). The predicted *Ae*. *aegypti* CDS were included with the hope of obtaining extensions of partial sequences. Indeed of the current 17,158 predicted proteins, 1,096 do not start with a methionine and 516 CDS do not finish with a stop codon. However, when the deducted proteins derived from the assembly were compared to the v3.3 *Ae*. *aegypti* proteome, we found 1,234 protein sequences that were extended by 25% or more in length. Although this appear to be a large number, when this subset of the v3.3 proteins are compared to the better annotated *D*. *melanogaster* proteome, including only blastp matches that have better e value than 1e-15, a total of 503 proteins have the *Drosophila* matches within 10% of the size of the assembly match. We additionally found 314 proteins that had matches covering over 90% of predicted *Culex pipiens quinquefasciatus* proteins but had less than 95% identity to *Ae*. *aegypti* v3.3 proteins. Although the purpose of the present work was not to extend the annotation of *A*. *aegypti*, we have manually confirmed the extension of 1,182 of these proteins and submitted their CDS to the TSA.

### Transcripts overexpressed in the salivary glands

**Fig 1:**
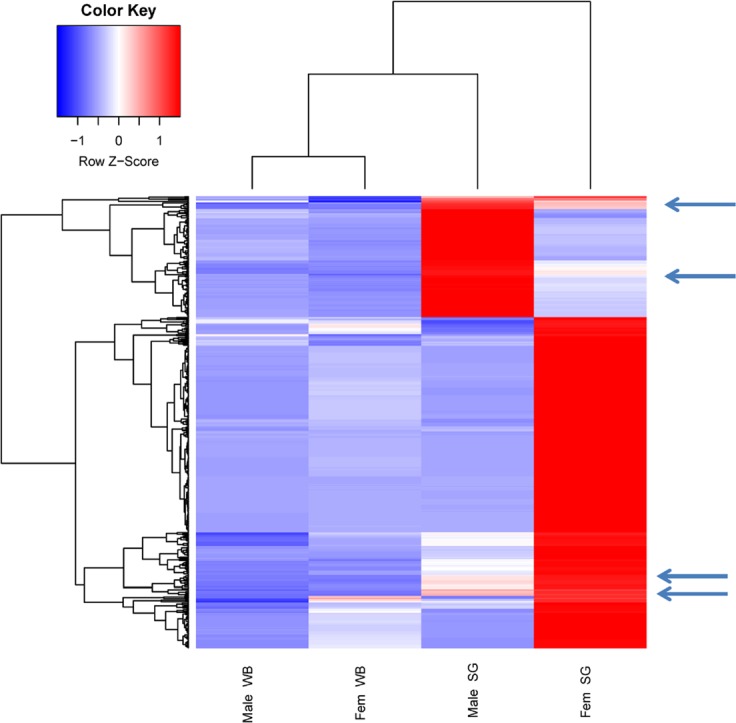
Differential gene expression in *Aedes aegypti*. Heat map of 10 fold upregulated salivary gland transcripts when compared to whole body, in male and female *Aedes aegypti*. The Z score transformed data of transcripts per million for each library is shown. The arrows point to some rows where expression on male glands is within 1 SD of female glands.

While exploring our data we aimed first to identify transcripts that were overexpressed in the total salivary gland set as compared to the whole body to obtain a set of products associated with salivary function independent of the insect gender. Next, within this salivary enriched group of transcripts we identified those arbitrarily fivefold or more overexpressed in either sex, and those within these boundaries. To identify transcripts that are overexpressed in the salivary glands we compared for each contig the sum of mapped reads derived from the salivary gland libraries with the sum of the reads derived from the whole body libraries, and performed a X^2^ test, the values of which were further corrected for multiple testing as indicated in the methods section. We further arbitrarily selected those transcripts that were at least 10 fold enriched in the salivary gland tissues, thus obtaining 360 transcripts, with the largest FDR value being 1.69 e-35 (S1 and S2 Files). Many of these transcripts are truncated, are possible splice variants or alleles, or may represent spurious open reading frames from anti-sense transcripts, but are all represented in S2 File for further confirmation, on worksheet “SG overexpressed”; a less redundant version without fragmented sequences is shown in the worksheet named “No fragments”. The heat map of these transcripts shows distinct clusters of male and female differentially expressed salivary gland transcripts, including transcripts that appear overexpressed in both male and female glands, as indicated by the arrows in Fig 1.

**Fig 2:**
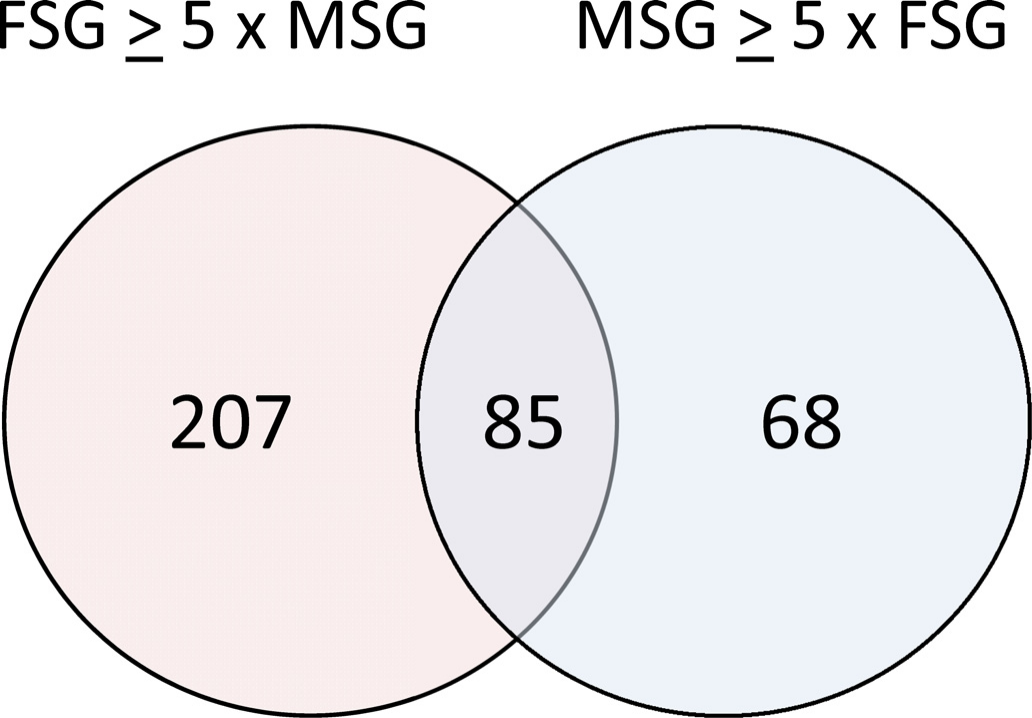
Sex-specific distribution of transcripts upregulated in the *Aedes aegypti* salivary glands. The 360 contigs found at least tenfold upregulated in the salivary glands when compared to whole bodies were partitioned in the three groups shown in the Venn diagram: FSG ≥ 5x MSG (overexpressed at least fivefold in FSG over MSG); MSG ≥ 5x FSG (overexpressed at least fivefold in MSG over FSG).

To further classify the transcripts overexpressed in the adult salivary glands, we partitioned the contigs in 3 categories: 1) Contigs that are overexpressed in FSG at least five fold over MSG, 2) Contigs that are overexpressed in MSG at least five fold over FSG, and 3) Contigs that are expressed in both male and FSG within a fivefold boundary (Fig 2).

To facilitate comparison of the current results with previous analysis, the S2 File also includes the results of expression studies from [24] (Column BA-BF of worksheet named “SG overexpressed”). This study compared transcript abundance by RT-PCR on FSG, female carcasses and whole adult males. Moreover, the lobe-specific localization of transcripts, as determined by [25] using an “in situ” hybridization protocol, is mapped to the same worksheet on column AZ. We have included these transcripts in our analysis even when they did not meet the 10 fold filter for SG overexpression over WB. We will make frequent reference to the EI, as well as to RPKM or TPM ratios indicative of selective tissue expression, as defined in the methods section.

#### Transcripts overexpressed in the female salivary glands–Introduction

We have found 207 transcripts that were overexpressed in the FSG. These transcripts are classified in three main categories, putative secreted, putative housekeeping and transposable elements (TE). The putative secreted class is further divided into enzymes, ubiquitous protein families, Nematocera- or mosquito-specific families and hypothetical secreted proteins (Table 1). The reader is here referred to our previous review on mosquito and nematoceran sialomes for understanding the protein family names and classification [1]. We will discuss the contigs expression levels determined in this study comparing it with previous work that used an RT-PCR approach [24] and considering salivary gland “in situ” RNA hybridization results from [25].

**Table 1:** Transcripts overexpressed at least fivefold in adult female *Aedes aegypti* salivary glands as compared to male glands. Transcripts per million average values deriving from reads obtained from male and female salivary gland libraries are shown (S2 File, columns HN and HM).

Class	Average Female TPM	Average Male TPM	Female /Male TPM	Number of contigs
**Putative secreted proteins**				
**Enzymes**				
Apyrase/5' nucleotidase	1,049.43	38.12	27.53	4
Adenosine deaminase	360.10	4.39	82.12	2
Purine hydrolase	1,145.78	55.31	20.72	3
Peroxidases	15.27	0.75	20.35	2
Phopholipase B	15.41	0.09	171.22	1
**Other ubiquitous families**				
Serpins	796.78	11.62	68.58	11
C-type lectins	859.91	7.31	117.61	7
Angiopoietin	621.39	6.70	92.81	2
His rich peptide	71.28	11.51	6.19	1
Antigen 5 related	1,304.42	25.97	50.22	12
**Mosquito specific families**				
D7 family related to the OBPs	2,368.28	38.52	61.48	20
Aegyptin	2,899.94	75.44	38.44	15
15–17 kDa family of blood feeding Diptera	179.46	1.87	96.23	2
9.7 kDa culicine family	1,573.40	46.72	33.68	7
12–14 kDa mosquito family	350.18	3.87	90.41	3
23.5 kDa culicine family	390.64	64.95	6.01	3
SG1/62/34 kDa superfamily	750.85	8.07	92.99	27
Sialokinin	1,572.47	12.88	122.07	7
4.2 kDa family	2,458.23	20.30	121.08	5
Proline rich peptide	189.23	1.49	127.00	3
gSG8 family	309.53	17.35	17.84	1
gSG5 protein family	150.97	5.26	28.69	4
8.7 kDa family	2,095.59	103.40	20.27	6
Mucins	937.28	24.79	37.82	4
Possible mucin	3,026.44	68.28	44.33	7
SGS family	261.33	3.38	77.32	19
**Hypothetical secreted proteins**	306.38	9.07	33.80	10
**Putative housekeeping proteins**				
Lipid metabolism	34.68	1.21	28.66	1
Signal transduction	20.11	2.46	8.17	1
Transcription factor	5.71	0.76	7.51	1
Conserved hypothetical proteins	8.33	1.24	6.72	1
**Hypothetical proteins**	1,159.45	50.26	23.07	13
Transposable elements	7.54	0.22	34.25	2
**Average**	827.14	21.93	55.01	
**Total**				207

#### Putative secreted transcripts overexpressed in the female salivary glands

Among the enzymes overexpressed in FSG, apyrase, 5’-nucleotidase, adenosine deaminase and purine hydrolase were found, as expected [23, 24, 39–42]. These have RPKM values larger than 500. The apyrase-coding gene AAEL006347 (EI = 40) had its expression previously mapped to the distal lateral lobes [25], as was AAEL006485 coding for the salivary purine hydrolase (EI = 23). AAEL005672, coding for the adenosine deaminase (EI = 12) was previously found enriched in FSG. Somewhat unexpectedly, we found a gene coding for a protein possessing a peroxidase domain, encoded by AAEL017467, which is only 24% identical at the amino acid level to the salivary peroxidase of *An*. *gambiae*. The annotated peroxidase gene, however, appears to be a fusion of a peroxidase gene and a Gypsy transposon. The 1,063-long predicted peptide matches the An_peroxidase CDD domain fully, from amino acid position 533–1,037. However, position 1–388 maps with 98% identity to the Gypsy-218_AA-I transposon as deposited in Repbase [43], and over 40% similarity to other *Aedes* Gypsy transposons. The reads mapped to AAEL017467 show low base coverage up to position 1500 (coinciding with the transposon domain), jumping to over 1,000 read coverage per base in the peroxidase domain, consistent with the absence of the transposon domain within the peroxidase gene product. A phospholipase B was also identified overexpressed 82 fold in FSG as compared to MSG (S2 File column AE), but it has low expression values (EI = 0.36 and RPKM = 13, S2 File columns AK and AC).

Regarding transcripts coding for proteins with ubiquitous domains, the three previously described salivary serpins (AAEL002704-PB EI = 32, AAEL003182-PA EI = 19 and AAEL007420-PB EI = 5) [23, 24], one of which is a factor Xa clotting inhibitor [44] were found, with some small sequence differences (~ 1% at amino acid level) from the reference v3.3 proteins. TPM ratios of FSG/MWB (S2 File column AI) are over 300–5,000 larger in FSG, and 20–160 fold when FSG values are compared to MSG (S2 File column AE). C-type lectins (AAEL000533-PA EI = 10 and AAEL000556-PA EI = 8) and angiopoietins (AAEL000749-PA EI = 9 and AAEL000726-PA EI = 11) were overtranscribed from 90–190 fold in female glands when compared to male glands, and several thousands on the FSG/MWB comparison. These transcripts have been found before to be FSG-specific by RT-PCR [24], and the C-type lectin transcript coded by AAEL000533 was mapped to the medial lobe [25]. Because these lectin-like proteins could be associated with immune function, and considering that immune related proteins such as lysozyme were previously found abundantly in both male and female glands [16, 45], there remained the doubt whether these lectin-like proteins were associated with blood feeding, but these results strongly indicate these lectins to be associated with blood feeding. A histidine rich peptide, that could be associated with immune function [46, 47], is only 5.5 times overexpressed in female glands, and perhaps is not associated with blood feeding. Antigen 5 proteins belong to the ubiquitous CAP family [48] and are widespread in insect and tick sialomes. Transcripts coding for the proteins AAEL003057-PA (EI = 3.3), AAEL003057-PB (EI = 4.5), AAEL003053-PC (EI = 23) and AAEL000793-PA (EI = 47) were found over 10 fold overexpressed in female glands, the last one being over 100 fold overexpressed (S2 File column AE), suggesting an unique association to hematophagy. The products of AAEL000793 and AAEL003053 were identified before on the distal lateral lobes [25]. AAEL000793-PA was also found to be FSG-specific by RT-PCR [24]. Interestingly, it will be shown below that there are other members of the same family that are overexpressed in MSG.

As expected, all D7 members of the mosquito proteome were found overexpressed in FSG, the long D7 protein AAEL006424-PA having the maximum EI = 100, as were the aegyptin family members (EI’s ranging from 20–87), in accordance with previous RT-PCR results [24] and “in situ” hybridizations [25]. Indeed FSG/MWB TPM ratios are over 1,000 in most cases and FSG/MSG are over 30. Many splice variants of the aegyptin family were found. The families 15–17 kDa (EI = 1.3), 9.7 kDa (EI = 12.4), 12–14 kDa (EI = 2.7), 23.5 kDa (EI = 4.2), SG1/62/34Kda (EIs ranging from 5 to 31.6), gSG5 (EI = 4.3), the vasodilator sialokinin (EI = 10.5), 4.2 kDa (EI = 11), gSG8 (EI = 3.3), 8.7 kDa (EI = 12.9), proline rich (EI = 2), and several mucins were also found highly overexpressed in female glands.

The 15–17 family was found previously by RT-PCR as expressed in female glands and in whole adult males and classified as expressed in both male and female glands (Ribeiro et al, 2007); our current results indicate an over 80 fold enrichment in FSG as compared to MSG, but the FSG/MWB TPM ratio is only 6, indicating that this transcript is expressed in some other(s) male tissue(s) and only to relative low level in MSG, thus explaining the discrepancy.

The 9.7 kDa family member AAEL008305, also designated as 7.8 kDa secreted protein, has over 30 fold higher expression in female over male SG as indicated by the TPM FSG/MSG ratio. It was previously mapped to the distal lateral and medial SG lobes [25] and found enriched in FSG [24]. It is highly expressed with RPKM’s ranging from 789–4,740 and an average EI = 7.9. The longer contig Ae-211362 reported in additional file 2 may represent an alternative spliced isoform. The 12–14 kDa family member AAEL009852 was previously classified as ubiquitous by RT-PCR (i.e. expressed in FSG but also in other female tissues and in whole adult males), and “in situ” hybridizations showed a weak staining of the medial lobe of female glands [25]. The FSG TPM values found here range from 340–369, for MSG it ranges from 3.6–4.3 but in female whole body (FWB) it ranges from 73 to 89 while in MWB it ranges from 0.02–0.05. Notice that the ratio of FSG to FWB is ~ 5, while the comparison for the more typical “female only” gene product, aegyptins, the ratio is over 100 when comparing FSG with FWB, and over 1,000 when comparing FSG to MWB. It is likely that some other female tissue may express this protein family, explaining the apparent discrepancy with the previous non quantitative RT-PCR result. Notice also from these comparisons that the TPM ratios FSG/MWB and MSG/FWB is quite instructive in detecting female or male SG specific gene products, as will be further discussed below.

Similarly, the 23.5 kDa family was previously indicated to be found in both female glands and adult males, and it is indeed borderline overexpressed in FSG, at 6 fold over MSG, but over 100 fold when FSG TPM are compared to MWB, indicating females may express this family elsewhere in addition to SG. Members of the 62/34 kDa family were shown before to be FSG specific or enriched by RT-PCR, and also expressed on distal lateral and medial lobes. Accordingly, their level of overexpression in FSG is over 50 fold over MSG (Table 1), and TPM ratios FSG/MWB are over 1,000.

The sialokinin and 4.2 kDa (AAEL008310) families were shown to be expressed in the medial lobe by “in situ” hybridization [25] and the latter shown to be FSG specific by RT-PCR [24]. The sialokinin gene, as well as the 4.2 kDa family member, are indeed 100 fold overexpressed in FSG versus MSG with EI of 10.5 and 11 respectively. The proline-rich peptide was also shown to be FSG specific by RT-PCR, and indeed it is over 100 fold overexpressed in FSG. The gSG8 product, however, was shown to be expressed in both SG genders by RT-PCR, but is 17 fold overexpressed in FSG when analyzed by the TPM ratio FSG/MSG. It also appears to be salivary enriched by the TPM ratio FSG/MWB = 337.

The gSG5, 8.7 kDa family, mucins and the SGS families were not previously analyzed for gender specific expression in *Ae*. *aegypti* and here are reported as being overexpressed in FSG, from ~ 20 fold (8.7 kDa family) to over 100 fold (some members of the SGS family). The SGS family was first identified in *An*. *gambiae* as a putative membrane protein, the gene of which was probably acquired via horizontal transfer [49]. They code for large proteins with more than 500 amino acids, the genes being mostly single exonic. Later, members of this family were identified as possible sporozoite receptors for SG invasion in *Aedes aegypti* [50]. Somewhat surprisingly, these proteins turned out to be found in saliva via a non-canonical secretory mechanism, where fragments of ~ 300 kDa are found in saliva and are major salivary immunogenic proteins [51]. Two members of this family were identified in the distal-lateral lobes of *An*. *gambiae* by immunocytochemistry, and to be overexpressed in FSG.

We additionally found 10 transcripts not previously annotated as salivary proteins, nor having matches to any known protein. Three of these code for peptides larger than 100 amino acids, the remaining code for smaller peptides that might derive from truncated transcripts or inverse open reading frames. AeSigP-210604 (EI = 5.8), AeSigP-195935 (EI = 2.3) and AeSigP-215268 (EI = 1.3), however, have FSG RPKM larger than 200 and expression ratios FSG/MSG over 90, and represent novel peptides without representation in the *Ae*. *aegypti* proteome and without similar proteins in GenBank or Refseq databases.

#### Putative housekeeping transcripts overexpressed in the female salivary glands

Relatively few transcripts associated with possible housekeeping function were found overexpressed in FSG. All have low expression values, with EI < 1, except for some hypothetical proteins of unknown function. AAEL000733 codes for a hydroxysteroid dehydrogenase, over 75 fold overexpressed in FSG as compared to MWB, and 26 fold overexpressed in FSG as compared to MSG. This enzyme may be associated with hormonal metabolism in FSG. Interestingly, the sex peptide receptor coded by AAEL010313 is also overexpressed in SG (FSG/MWB = 10), and more so in FSG (FSG/MSG = 7.8). The doublesex transcription factor (Ae-3256) is also similarly overexpressed in the SG (FSG/MWB = 14) and in the FSG (FSG/MSG = 6.6). Additional gene products overexpressed in FSG include several hypothetical proteins, a few with relatively high EI and SG overexpression values (AeSigP-215960, EI = 38, FSG/MWB = 10,533 and AeSigP-209690, EI = 45, FSG/MWB = 9,400).

#### Putative transposable elements overexpressed in the female salivary glands

Two transposable elements were overexpressed in FSG, both of which appear more than 20 fold overexpressed in FSG as compared to MSG, although their EI are low (0.27 and 0.61). AeTE-198758 codes for a protein with 1,363 amino acids having the CDD domain AIR1 coding for arginine methyltransferase-interacting protein from aa position 192–304, a retropepsin_like domain, which are pepsin-like aspartate proteases from retroviruses, from 373–464, a RT_LTR domain (evalue = 2 e -51) coding for reverse transcriptase from 536–712 and aRNase_HI_Ty3 motif from 807–929. It is 99% identical over 1363 amino acids to element TF000143 deposited in TEFAM and to Gypsy-296_AA-I deposited in Repbase. Importantly, similar elements were also found in deep sequenced sialotranscriptomes of adult female *Psorophora albipes* [28] and the frog feeding fly *Corethrella appendiculata* [29], indicating these transposons to be widely expressed in the sialotranscriptomes of Culicomorpha. The additional element matches > 98% of Repbase-deposited element BEL-6_AA-I [43], and best matches also another transposon derived from the sialotranscriptome of *C*. *appendiculata*.

### Transcripts overexpressed near equally in adult salivary glands

Here we describe those transcripts that are expressed over 10 fold in the SG as compared to WB, but no more than 5 fold overexpressed when comparing FSG to MSG or vice versa. A total of 85 transcripts were thus found, classified as putative secreted proteins, putative housekeeping proteins, transposable elements and viral products (Table 2).

**Table 2:** Transcripts similarly expressed in male and female salivary glands of adult *Aedes aegypti* mosquitoes (within 5 fold difference). Transcripts per million average values deriving from reads obtained from male and female salivary gland libraries are shown (S2 File, columns HN and HM).

Class	Average Female TPM	Average Male TPM	Female /Male TPM	Number of contigs
**Putative secreted proteins**				
**Enzymes**				
Alkaline phosphatase	59.17	63.81	0.93	1
Lipase	6.35	11.43	0.56	1
Amylase	363.71	853.88	0.43	3
Serine proteases	567.02	246.15	2.30	8
**Immunity related**				
Gram-negative binding protein	488.43	789.81	0.62	2
TIL domain containing peptide	83.54	326.64	0.26	2
Lysozyme	1,996.11	4,780.93	0.42	4
Gambicin	345.05	442.82	0.78	2
**Small molecule binding protein**				
Phosphatidylethanolamine-binding protein	21.32	47.00	0.45	1
**Mucins**	78.05	106.48	0.73	9
**Mosquito specific families**				
41 kDa family	225.64	802.93	0.28	3
16.6 kDa family	3,553.76	1,501.07	2.37	5
7.9 kDa family	851.27	3,237.94	0.26	4
Conserved secreted proteins	52.13	18.46	2.82	3
**Hypothetical secreted proteins**	814.02	512.80	1.59	12
**Putative housekeeping proteins**				
Amino acid metabolism	28.58	15.51	1.84	1
Protein modification	33.16	10.08	3.29	1
**Hypothetical proteins**	882.09	397.77	2.22	11
**Transposable elements**	19.48	7.08	2.75	3
**Viral products**	85.10	39.79	2.14	9
**Average**	527.70	710.62	1.35	
**Total**				85

#### Putative secreted transcripts overexpressed near equally in adult salivary glands

The putative secreted category was similarly classified as enzymes, immune-related, small molecule binding, mucins, mosquito specific families, conserved secreted proteins and hypothetical secreted proteins.

Previously identified transcripts analyzed by RT-PCR [24] or in-situ RNA hybridization [25] and deemed to be sex unspecific were found in this category. These include the gene products coding for serine proteases, amylases, Gram-negative binding protein, lysozyme, TIL-domain containing peptides, gambicin, 41 kDa family, and 16.6 kDa family.

An alkaline phosphatase (EI = 1.8), only 2 fold overexpressed in SG when compared to WB, and a lipase (EI = 0.1, 10 fold overexpressed in SG) were found similarly expressed in both MSG and FSG, indicating these enzymes may play a role unrelated to blood feeding, as is with the shared amylase gene (EI = 23.3), which is 12 fold overexpressed in the SG when compared to WB, but the ratio FSG/MSG is only 0.4. The immunity related products Gram-negative binding protein (EI = 11.4), lysozyme (EI = 18.2, 11 x overrepresented in the SG) and gambicin (EI = 1.7, only 1.1 x overrepresented in the SG) were expected to be represented in both male and female SG, as they may help to control bacterial growth in the crop stored sugar meals. Peptides containing the Trypsin-Inhibitor Like (TIL) domain can also function as antimicrobials. They were found in *An*. *gambiae* female sialotranscriptomes [49, 52] and later on in male sialotranscriptomes of the same mosquito [53]. The gene coding for gi|94468538 (v3.3 not containing it) is 8 fold overexpressed in the SG with an EI = 0.4. The mosquito specific family 41 kDa (AAEL004382-PA, EI = 5.8, SG/WB = 12.6) was also found in male *An*. *gambiae* sialotranscriptomes, as well as in the non-blood feeding mosquito *Toxorhinchites amboinensis* [54]. The gene products encoding for the 16.6 kDa protein (AAEL007986-PA, EI = 47.4, FSG/MWB = 13, previously found expressed in the proximal lateral lobes) and 7.9 kDa family (gi|65306522, not on v3.3, EI = 5, MSG/FWB>40) as well as for several mucins, conserved secreted proteins and hypothetical proteins are salivary-enriched but gender unspecific. This includes the contig coding for AAEL009194-PA (EI = 0.25, SG/WB = 3) which was previously characterized as female specific, the only major discrepancy between this study and the RT-PCR results previously reported [24].

#### Putative housekeeping transcripts overexpressed near equally in adult salivary glands

Transcripts coding for kynurenine formamidase and a DnaJ chaperone were identified as been overexpressed in adult SG as compared to WB. Kynurenine formamidase catalyses the second step of degradation of the amino acid tryptophan, from N-formyl-L-kynurenine to L-kynurenine. Interestingly, L-kynurenine can be converted by arylformamidase to 3-Hydroxy-L-kynurenine which can be converted by kynurenine-oxoglutarate transaminase to xanthurenic acid, a compound previously found in mosquito salivary glands and associated with *Plasmodium* male gametocyte exflagellation [55–57]. Several hypothetical proteins with a possible housekeeping role were also identified as enriched in both male and female SG.

#### Putative transposable elements and viral-derived transcripts overexpressed near equally in adult salivary glands

Three contigs coding for Transposable elements were identified as overexpressed 20–100 fold in SG as compared to WB. Two of these match different regions of Gypsy-240_AA-I from Repbase [43], and the third matches with 52% identity a 154 amino acid stretch of Gypsy-591_AA-LTR, also from Repbase.

In addition to transposons, nine contigs were found coding for viral-like proteins. Ae-197779 does not match any predicted Ae. *aegypti* protein, but matches the genomic encoded cell fusing agent virus polyprotein-like protein of *Ae*. *albopictus* with 39% identity over a 1,130 amino acid stretch [58], and also the polyprotein precursor of Kamiti River virus [59], again with 39% identity over a 1,127 amino acid stretch. The predicted protein has a CDD Flavivirus DEAD domain preceded by a PFAM peptidase _S7 typical of Flavivirus NS3 protease. Ae-200358, AeSigP-212951 and Ae-206778 appear to be fragments of the same virus transcript, all matching with 35–56% identity the protein coded by gi|577735311 and annotated as Replicase large subunit of *Ceratitis capitata*. These fragments have the PFAM domain RdRP_2, RNA dependent RNA polymerase (full domain, evalue=2e-72), as well as the full PFAM domain pfam01443, Viral_helicase1 (evalue = 64e-73). The ratios of SG to WB being over 1,500, of the same order as uniquely female gland expressed proteins such as aegyptin, salivary serpins or the D7 members. Ae-200529 produces a match to AAEL002535-PA, but having only 84% identity. This product has the PFAM domain, Rhabdo_ncap, coding for Rhabdovirus nucleocapsid protein. Most of these viral transcripts map to supercontig1.286, but also to supercontig1.1, 1.20 and 1.1145.

### Transcripts overexpressed in adult male salivary glands

Sixty eight transcripts were found overexpressed fivefold or more in MSG when compared to female glands, the average expression ratio being male TPM/female TPM = 17.52 (Table 3). Notice that the reverse comparison had a female TPM/Male TPM = 55 (Table 1). As above, we classify these male overexpressed transcripts in putative secreted proteins, putative housekeeping proteins and transposable elements.

**Table 3:** Transcripts overexpressed at least five fold in *Aedes aegypti* male salivary glands as compared to female glands. Transcripts per million average values deriving from reads obtained from male and female salivary gland libraries are shown (S2 File, columns HN and HM).

Class	Average Female TPM	Average Male TPM	Female /Male TPM	Number of contigs
**Putative secreted proteins**				
**Enzymes**				
Lipases	40.85	0.20	204.25	1
Maltase	7,620.96	849.62	8.97	4
Serine proteases	889.03	144.83	6.14	6
**Other ubiquitous families**				
Antigen 5 related	3,985.90	576.58	6.91	2
Gram negative binding protein	251.20	35.84	7.01	1
TIL domain containing peptide	21.13	4.11	5.14	1
**Mosquito specific families**				
Nematocera mucin I family	3,139.52	122.92	25.54	5
8.3 kDa mucin family	25,486.79	3,514.27	7.25	8
SG3 mucin	8,576.15	728.76	11.77	5
Other mucin	415.27	76.95	5.40	1
56 kDa family	8,357.64	1,531.35	5.46	1
30.5 kDa family	3,572.05	457.08	7.81	5
hyp10/hyp12 anopheline type family	2,326.55	361.96	6.43	8
7.1 kDa His rich peptide	15,541.50	2,299.07	6.76	4
W rich peptide	5,755.65	1,139.71	5.05	2
Hyp 6.2 kDa superfamily	236.34	34.58	6.83	1
**Hypothetical secreted proteins**	6,741.66	908.24	7.42	6
**Putative housekeeping proteins**				
Signal transduction	93.93	12.79	7.34	1
Ion regulation	80.98	6.58	12.31	1
**Hypothetical proteins**	5,932.60	790.59	7.50	3
**Transposable elements**	1,153.83	175.79	6.56	2
**Average**	4,772.36	655.80	17.52	
**Total**				68

#### Putative secreted transcripts overexpressed in adult male salivary glands

Previously the genes coding for maltase (AAEL000392) and two serine-proteases (AAEL015294 and AAEL005596) were shown by “in situ” hybridization to express their transcripts primarily in the proximal lateral lobes [25]. The protease product of AAEL005596 was also shown by RT-PCR to be found in both female SG and adult males. Additional “in situ” studies identified the products coding for the 56 kDa protein (AAEL009081) and W-rich peptide (AAEL004597) to be expressed in the proximal lateral lobes [25]. Previous RT-PCR studies identified the antigen-5 coding product of AAEL002693, the Gram-negative binding product of AAEL003889, the TIL-domain containing peptide coded by AAEL005487, the SG3 mucin (gi|94468426), the hyp10/hyp12 family member gi|94468396, the 7.1 kDa peptide, and the hyp 6.2 kDa peptide as expressed in both female glands and adult males [24]. The putative 30.5 kDa member coded by AAEL007780, however, was found enriched, but not exclusively found in female glands by RT-PCR, while we found it 11 fold enriched in male glands (Table 3 and additional file 2).

We additionally identify here male enriched transcripts coding for a lipase encoded by AAEL000828, which is wrongly annotated as vitellogenin, probably because this egg protein also has a lipase domain. Notably this product is expressed 136 fold more in MSG, being potentially the best marker of male salivary glands. Several mucins appear also to be quite male specific, AAEL012423 from the Nematocera mucin I family and alleles being 23–31 fold overexpressed, while the 8.3 kDa mucin family members are only 7–8 times overexpressed. Six hypothetical secreted proteins are 5–10 fold enriched in MSG.

#### Putative housekeeping transcripts overexpressed adult male salivary glands

A few contigs coding for putative housekeeping proteins were identified to be overexpressed in MSG, ranging from 6–20 fold enrichment. These include contigs coding for a novel PDZ domain containing protein and three hypothetical proteins. We include here also the gene coding for carbonic anhydrase (AAEL010893), which is 12 fold overexpressed in MSG in relation to FSG, although the overall SG to WB expression is only 6 fold, and was previously found expressed in the proximal-lateral lobes [25]. The other housekeeping contigs vary from 5–22 fold overexpression on MSG compared to FSG.

The novel PDZ domain-containing protein is 240 fold enriched in salivary glands as compared to whole body, having a relatively high RPKM of 388 in MSG and of 47 in FSG, but only 3.2 and 0.5 in MWB and FWB, respectively. It has no matches to known *Ae*. *aegypti* proteins in Vector Base or GenBank, but is 90% identical to a 652 amino acid stretch of a *C*. *pipiens quinquefasciatus* protein of 745 amino acids and matches *D*. *melanogaster* Patj homolog, which is expressed in primary and some secondary epithelial cells such as salivary glands, foregut and hindgut [60].

#### Putative transposable elements overexpressed in adult male salivary glands

Two class I transposable elements were overexpressed in MSG. Both, coded by Ae-209592 and Ae-183933, are > 97% identical at the amino acid level to Repbase Gypsy-5_AA-I and Gypsy-164_AA-I, respectively [43]. These elements are only 6–7 fold overexpressed in MSG when compared to FSG, but are 74–1,700 fold overexpressed in MSG when compared to FWB (or 20–1,300 fold overexpressed in SG when compared to WB).

### Transcripts with extreme salivary overexpression in relation to body tissues

We draw attention to the TPM ratios derived from SG and WB libraries. As an example, the transcript coding for the enzyme adenosine deaminase has the following TPM ratios (S2 File, columns AE-AH): FSG/MSG = 77, FSG/FWB = 7.5, FSG/MWB of 131 and FWB/MWB = 17 indicating transcript predominance in female tissues and more so in the salivary glands. The male ratios show MSG/MWB = 2.83 and MSG/FWB = 0.17. Comparing the same values deriving from the transcript coding for the salivary serpin we obtain: FSG/MSG = 108, FSG/FWB = 8.45, FSG/MWB = 5,132 and FWB/MWB = 607, and for the male libraries, MSG/MWB = 46, MSG/FWB = 0.08. Notice the large FSG/MWB value indicative of extreme salivary female abundance. It appears that while adenosine deaminase is enriched in FSG, the serpin in much more so, indicating the enzyme may be expressed in many more additional adult tissues than the serpin product. Table 4 and S2 File, worksheet “Salivary specific” show the transcripts that are thus most abundant in female glands and having a FSG/MSG > 1,000.

**Table 4:** Transcripts with extreme salivary overexpression in relation to body tissues. Salivary products extremely overexpressed in relation to whole body, as determined by the transcripts per million ratios obtained from male (M) and female (F) salivary gland (SG) and whole body (WB) libraries having values above 1,000.

Class	Average TPM FSG/ (MSG+0.1)	Average TPM FSG/ (MWB+0.1)	Average TPM MSG/(FWB+0.1)	N
**Female overexpressed**				
Serpins	78.82	2,539.95	0.13	3
C-type lectins	116.62	8,947.54	0.24	3
Angiopoietin	91.35	4,390.17	0.12	2
Antigen 5 related	121.86	2,005.36	0.23	1
D7 family	77.89	6,161.07	0.09	8
Aegyptin	44.55	1,704.06	0.35	1
9.7 kDa culicine family	33.95	1,155.48	0.88	1
12–14 kDa family	91.94	2,420.71	0.05	1
SG1/62/34 kDa superfamily	101.99	1,644.67	0.19	6
Sialokinin	121.86	9,543.28	0.05	2
4.2 kDa family	121.59	15,302.12	0.42	1
Mucins	87.85	1,993.93	0.31	4
Possible mucin	43.61	5,322.81	1.48	7
SGS family	91.48	1,166.29	0.11	2
Hypothetical secreted proteins	109.99	6,842.89	0.08	1
Hypothetical proteins	59.35	6,388.39	2.23	4
**Male similar to female**				
Hypothetical secreted proteins	2.30	3,479.42	1,515.11	2
Viral products	2.12	2,346.32	1,112.10	3
**Male overexpressed**				
Hypothetical protein	0.17	2,741.81	20,730.31	1
Transposable element	0.15	560.68	1,710.26	1
**Total**				54

### qPCR validation of RPKM ratio data

**Fig 3:**
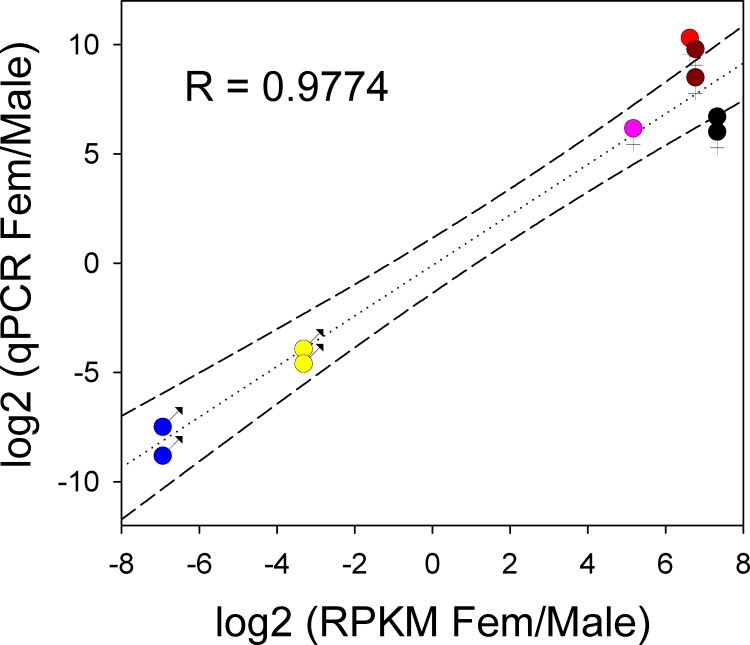
Female to male differential gene expression as determined by RNASeq or qPCR. The graph represents the log2 fold ratio of female to male expression of six differentially expressed genes. Female enriched transcripts have the female symbol and the colors identify the following genes: Red: Sialokinin Ae-207373, dark red: 4.2 kDa peptide Ae-207907, black: phospholipase b Ae-206009 and pink: aegyptin Ae-211729. Male enriched transcripts have the male symbols and the colors identify the following genes: Yellow, glycosidase Ae-195553 and blue: lipase Ae-212377. The genes shown in duplicate (all except sialokinin and aegyptin) were done with different primer pairs (S1 Table). All qPCR experiments were done with three biological replicates each done in duplicate instrument measurements. The lines represent the linear regression and its 95% confidence intervals.

Because our experimental design included a single replicate for each gender-derived salivary gland library, we sought to validate the results using qPCR for selected transcripts, including contigs overexpressed in female (Sialokinin Ae-207373, 4.2 kDa peptide Ae-207907, phospholipase b Ae-206009 and aegyptin Ae-211729) as well in male (glycosidase Ae-195553 and lipase Ae-212377). Results (S1 Table and Fig 3) indicated a high correlation of the independently estimated ratios (R = 0.97, p<0.001) and a possible underestimation of the RPKM ratios when compared to the qPCR-derived ratios. For example, the FSG/MSG ratio for sialokinin and 4.2 kDa peptides were 1.7–11 times higher by qPCR (60–1069 fold) than by RPKM (36–109 fold), and the MSG/FSG ratios for the male enriched glycosidase (12–20 fold) and lipase (151 and 377 fold) were 1.4–3.4 larger by qPCR when compared to the RPKM derived ratios.

## Conclusions

The application of RNASeq data from adult male and female salivary glands, combined with whole body data of both sexes allowed for an insight into what products are unique to salivary glands, and which of them are gender specific. Although there were no biological replicates used, the resulting analysis is highly consistent with previous work using tissue-specific RT-PCR studies [24] and female salivary gland “in situ” hybridization studies [25], thus supporting the conclusions drawn. We additionally verified by qPCR the gender expression ratios of six selected genes, which were in agreement with the RPKM-derived ratios. We also call attention to the importance of publicly available RNASeq data [27] that was important for this work.

While much has been done in uncovering the role of salivary proteins of mosquitoes in feeding and in particular how they affect host hemostasis, immunity and inflammation [1, 61, 62], we highlight the unknown function of the C-type lectins, angiopoietins, female-specific antigen 5 protein, the 9.7 kDa, 12–14 kDa, 23.5 kDa, 62/34 kDa, 4.2 kDa, proline rich peptide, gSG8, 8.7 kDa family and SGS fragments, which we have no idea of their function yet. They are overexpressed in FSG, are putatively secreted and should affect host physiology.

Although proteases have been found associated with salivary fibrinolytic activity in ticks and horse flies, the expression of salivary serine proteases in *Ae*. *aegypti* appear to be gender independent, or even overexpressed in male SG, indicative of a non-blood feeding role. The salivary phosphatase and lipase can also be interpreted in the same manner. Fewer transcripts were found enriched in male salivary glands when compared to the opposite sex transcriptome, and in this case the enrichment was mild in comparison to female enriched transcripts. This result is expected by the overlap of the sugar feeding mode on both sexes and the uniqueness of blood feeding in females. It is actually surprising the level of male expression of a lipase found ~200 fold enriched in MSG when compared to FSG (confirmed by qPCR, S1 Table and Fig 3). Can this lipase release some product functioning as a kairomone when male mosquitoes feed?

Only one transcription factor, “doublesex”, was identified as salivary female gland overexpressed. Doublesex is expressed as different spliced versions in male and female organisms and control differential sexual expression of many genes [63, 64] and may be affecting salivary gland differentiation and dimorphism. Perhaps differential expression of transposable elements in male, female and gender unspecific salivary glands may regulate gene expression in these organs, as the role of non-coding RNAs in regulating gene expression emerges [65–67]. Notably, homologous elements have been found following adult FSG RNAseq of *Psorophora albipes* and *Corethrella appendiculata*, indicating the conservation of the expression of these elements in the SG of Culicomorpha over hundreds of millions of years. RNAi experiments interfering with selected TE expression may uncover their role in regulating gene expression in mosquito SG.

Several endogenous viral transcripts, probably acquired by horizontal transfer, were also found expressed uniquely in the SG, without much sex bias, and may represent a mechanism of virus interference.

Finally, as we walk over a new methodological threshold within the scope of RNAseq, it cannot be overemphasized the amount and quality of this newly generated data when properly assembled and annotated, as compared to previously available Sanger-based EST’s. While the main purpose of this work was to identify gender-specific salivary transcripts, we were able to extend hundreds of genes and identify new ones, something not possible with microarray techniques that rely on previously predicted sequences.

## Supporting Information

S1 File Hyperlinked excel spreadsheet with reassembled coding sequences and reads mapped with RPKM > 5. (ZIP)

S2 File Hyperlinked spreadsheet with salivary differentially expressed transcripts. (ZIP)

S1 Table Primers utilized for qPCR studies. (XLSX)
